# Aerobic, resistance, or combined exercise training and cardiovascular risk profile in overweight or obese adults: the CardioRACE trial

**DOI:** 10.1093/eurheartj/ehad827

**Published:** 2024-01-17

**Authors:** Duck-chul Lee, Angelique G Brellenthin, Lorraine M Lanningham-Foster, Marian L Kohut, Yehua Li

**Affiliations:** Department of Kinesiology, Iowa State University, 534 Wallace Road, Ames, IA 50011, USA; Department of Kinesiology, Iowa State University, 534 Wallace Road, Ames, IA 50011, USA; Department of Kinesiology, Iowa State University, 534 Wallace Road, Ames, IA 50011, USA; Department of Kinesiology, Iowa State University, 534 Wallace Road, Ames, IA 50011, USA; Department of Statistics, University of California-Riverside, Riverside, CA, USA

**Keywords:** Aerobic, Resistance, Combined exercise, Cardiovascular disease, Obesity

## Abstract

**Background and Aims:**

To determine the comparative efficacy of resistance, aerobic, and combined resistance plus aerobic exercise on cardiovascular disease (CVD) risk profile.

**Methods:**

This randomized controlled trial enrolled 406 adults aged 35–70 years with overweight or obesity and elevated blood pressure. Participants were randomly assigned to resistance (*n* = 102), aerobic (*n* = 101), combined resistance plus aerobic exercise (*n* = 101), or no-exercise control (*n* = 102). All exercise participants were prescribed 1 h of time-matched supervised exercise (the combination group with 30 min of each resistance and aerobic exercise) three times per week for 1 year. The primary outcome was the change from baseline to 1 year in the standardized composite *Z*-score of four well-established CVD risk factors: systolic blood pressure, low-density lipoprotein (LDL) cholesterol, fasting glucose, and per cent body fat.

**Results:**

Among 406 participants (53% women), 381 (94%) completed 1-year follow-up. Compared with the control group, the composite *Z*-score decreased at 1 year, which indicates improved CVD risk profile, in the aerobic {mean difference, −0.15 [95% confidence interval (CI): −0.27 to −0.04]; *P* = .01} and combination [mean difference, −0.16 (95% CI: −0.27 to −0.04); *P* = .009] groups, but not in the resistance [mean difference, −0.02 (95% CI: −0.14 to 0.09); *P* = .69] group. Both aerobic and combination groups had greater reductions in the composite *Z*-score compared with the resistance group (both *P* = .03), and there was no difference between the aerobic and combination groups (*P* = .96). Regarding the four individual CVD risk factors, only per cent body fat decreased in all three exercise groups at 1 year, but systolic blood pressure, LDL cholesterol, and fasting glucose did not decrease in any exercise groups, compared with the control group.

**Conclusions:**

In adults with overweight or obesity, aerobic exercise alone or combined resistance plus aerobic exercise, but not resistance exercise alone, improved composite CVD risk profile compared with the control.

## Structured Graphical Abstract

**Structured Graphical Abstract:**
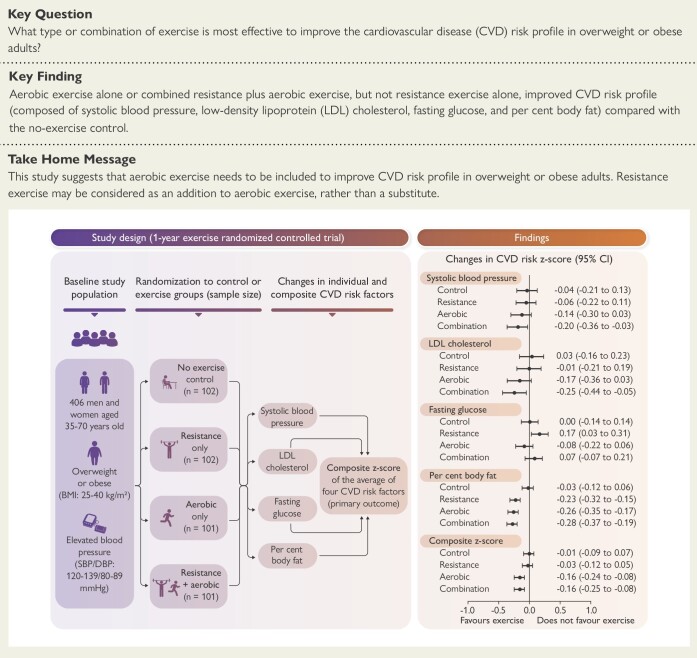
Effects of 1-year resistance, aerobic, or combined exercise training on cardiovascular disease (CVD) risk profile: the CardioRACE trial. Aerobic exercise alone or combined aerobic plus resistance exercise, but not resistance exercise alone, improved CVD risk profile (composite *Z*-score) compared with no-exercise control (*Z*-score values below 0 indicate favourable changes in CVD risk factors). BMI, body mass index; CI, confidence interval; CVD, cardiovascular disease; DBP, diastolic blood pressure; SBP, systolic blood pressure.

**See the editorial comment for this article ‘A new dawn of managing cardiovascular risk in obesity: the importance of combining lifestyle intervention and medication', by M. Halle and M. Papadakis, https://doi.org/10.1093/eurheartj/ehae091.**

## Introduction

Cardiovascular disease (CVD) is the leading cause of death, accounting for approximately one-third of all deaths in the USA and globally.^1,2^ Physical activity, particularly aerobic exercise, has been well established to prevent CVD.^3–5^ However, few clinical trials have directly tested if resistance or combined resistance plus aerobic exercise provides similar or stronger cardiovascular benefits compared with aerobic exercise alone. Therefore, one of the commonly asked questions, ‘What type or combination of exercise is most effective to prevent CVD?’, remains unanswered, especially in populations with overweight or obesity who are at increased risk of CVD.^6^

Different exercises affect different physiological systems and functions related to multiple CVD risk factors. Aerobic exercise generally improves haemodynamics, lipid profile, and cardiorespiratory fitness, whereas resistance exercise improves glucose metabolism, body composition (e.g. lean mass), and muscular strength.^7^ Most individuals show improvements in some, but not all, CVD risk factors in response to exercise. Thus, focusing on a single CVD risk factor, which has been the approach in most exercise studies, may not fully capture the comprehensive and comparative effects of different types of exercise on overall CVD risk profile. Individuals who develop CVD typically have a cluster of major CVD risk factors such as hypertension, dyslipidaemia, diabetes, and obesity, and the presence of multiple risk factors increases CVD risk.^8,9^ Therefore, to assess and predict future CVD, composite CVD risk scores (e.g. Framingham or atherosclerotic CVD risk scores) are widely used in clinical practice, yet are not usually applied in clinical trials.^10,11^ Considering these factors, examining composite CVD risk scores is clinically useful, especially when comparing the overall cardiovascular effects of various types of exercise.

Some trials demonstrated the superior health benefits of combined resistance plus aerobic exercise than either exercise alone on frailty in older adults and type 2 diabetes,^12,13^ although limited data exist on CVD. However, the combined exercise groups often performed twice the exercise time than either exercise alone. Thus, it is unclear whether the superior benefits in the combined exercise were due to extra exercise time, which is a considerable limitation given that lack of time is the foremost barrier to exercise from a public health perspective. Therefore, this study was designed to compare the effects of 1-year, time-matched resistance, aerobic, and combined resistance plus aerobic exercise on CVD risk profile, specifically using time to equalize the total volume of exercise (i.e. 60 min/session) between exercise groups for practical and direct public health implications. The CVD risk profile was comprised of the four major traditional, yet modifiable, CVD risk factors: systolic blood pressure, LDL cholesterol (LDL-C), fasting glucose, and per cent body fat.^14^

## Methods

### Study design

Comparison of the Cardiovascular Benefits of Resistance, Aerobic, and Combined Exercise (CardioRACE) study was a randomized controlled trial, conducted from July 2017 through March 2020 at Iowa State University. The protocol was approved by the institutional review board, monitored by an independent data and safety monitoring board, and published earlier.^15^

### Participants

Participants were recruited through various strategies including advertisements and mass mailings. Participants underwent initial eligibility screening based on measured body mass index (BMI) and blood pressure and participated in a month-long run-in period to learn study procedures and ascertain their ability to adhere to the 1-year intervention before randomization. Participants were eligible if they were non-smokers, 35–70 years old, inactive over the past 3 months (not meeting the current both aerobic and resistance exercise guidelines, which are ≥150 min/week of moderate or ≥75 min/week of vigorous aerobic exercise and ≥2 days/week of resistance exercise),^3^ overweight or obese (BMI 25–40 kg/m^2^), and had elevated blood pressure (systolic/diastolic blood pressure 120–139/80–89 mmHg) without taking antihypertensive medications. Major exclusion criteria were CVD (e.g. myocardial infarction and stroke), cancer, and severe arthritis that precluded exercise training. Full eligibility criteria were described previously.^15^ All participants provided written informed consent.

### Randomization

After the run-in period, participants completed baseline assessments and then were randomly assigned by the masked study statistician to one of four groups: resistance, aerobic, combined resistance plus aerobic, or no-exercise control, using randomized permuted block designs stratified by race/ethnicity (non-Hispanic white or all other races and ethnicities), sex, age (35–44, 45–54, 55–64, or 65–70 years), and BMI (25–29, 30–34, or 35–40 kg/m^2^). The study statistician was not involved in participant recruitment, exercise intervention, or outcome assessments. The exercise intervention staff were excluded from outcome assessment, and the outcome assessment staff were excluded from the exercise intervention and blinded to the group assignments. Participants were instructed not to reveal their group assignment during outcome assessments.

### Interventions

The exercise intervention was developed based on the American College of Sports Medicine (ACSM) and the US and WHO Physical Activity Guidelines^3,4,16^ as well as the exercise programmes proven to be feasible in large trials.^13,17^ The control group did not come to the lab to exercise during the first year, but were offered 1 year of delayed exercise (i.e. in Year 2) to prevent dropout. All exercise sessions were prescribed three times per week for 60 min and consisted of 5 min of warm-up, 50 min of resistance training for the resistance group, 50 min of aerobic training for the aerobic group, or 25 min of resistance plus 25 min of aerobic training for the combination group, and 5 min of cooldown. The resistance group was prescribed three sets of 8–16 repetitions at 50%–80% of one-repetition maximum (1RM; the maximum weight a participant can lift in one attempt) on 12 weight-lifting machines (leg press, hamstring curl, quadriceps extension, hip abduction, chest press, lat pulldown, shoulder press, biceps curl, triceps extension, abdominal crunch, lower back extension, and torso rotation) with ∼1 min of rest between sets and machines. Participants were asked to complete each set to fatigue, so they could not lift more than their prescribed repetitions. The aerobic group was prescribed exercise at 50%–80% of their heart rate reserve (HRR), calculated as ‘[(maximum heart rate − resting heart rate) × %intensity] + resting heart rate’ (where 0% is resting and 100% is maximal effort)^3,16^ using treadmills, stationary bicycles, and elliptical machines. The combination group was prescribed aerobic exercise at the same 50%–80% HRR and two sets of 8–16 repetitions at 50%–80% of 1RM on nine machines (excluding hip abduction, biceps curl, and triceps extension) to achieve half of the total sets compared with the resistance group. Each exercise session was prescribed to start at 50%–60% of 1RM or HRR, and then exercise intensity was increased to target at least half of exercise at 60%–65% of 1RM or HRR, although participants were allowed to exercise up to 80% of 1RM or HRR, following the recommended moderate-to-vigorous intensity exercise guidelines.^3,4,16^ We re-evaluated each individual’s 1RM and HRR every 2 months to update the exercise prescription, potentially maximize exercise benefits, prevent boredom, and motivate participants continuously throughout 1 year.

All exercise programmes were individually prescribed considering fitness levels, health conditions, adaptation to prior sessions, and progression; supervised by trained research staff; and recorded automatically using a computer-controlled training system (Technogym Wellness System).^15^ For example, participants wore a heart rate monitor on their chest, and each aerobic machine adjusted its intensity (e.g. treadmill speed and grade) automatically to keep participants within the prescribed HRR. Participants followed individually programmed sets, repetitions, and weights on each weight-lifting machine. After each exercise, detailed exercise parameters (e.g. heart rate and weight lifted) were automatically stored in the computer for more accurate exercise adherence calculation.

All participants were asked to maintain their usual lifestyle physical activity outside of exercise sessions. We measured daily steps using an accelerometer-based pedometer (Omron HJ-321) and weekly muscle-strengthening activities using self-report throughout 1 year in all participants including the control group. Although healthy diet and exercise are commonly suggested together for CVD prevention,^14^ diet is often underappreciated in exercise trials. In this study, all participants including the control group received the same Dietary Approaches to Stop Hypertension (DASH) diet education (e.g. reduced sodium intake)^18^ during the run-in period and counselling with a registered dietitian every 3 months to promote a standardized, CVD prevention eating approach. The DASH diet education focused on diet quality, but was neither a calorie reduction nor diet intervention programme for weight loss in this study. All participants were asked to complete a 24-h food recall on three random days per month (two weekdays and one weekend day) for 1 year using the Automated Self-Administered 24-Hour (ASA24) Dietary Assessment Tool, developed by the US National Cancer Institute.^19^

To minimize dropout, we employed various strategies including motivational interviewing, behavioural contract signing, flexible exercise scheduling, upper (e.g. rotator cuff) and lower body (e.g. knee) injury prevention exercises, and regular exercise adherence reports. All participants received monthly phone calls (called ‘I care calls’) to discuss their study experience and solve any issues. Each participant was given up to $300 over 1 year as an incentive, similar to early efficacy studies.^17,20,21^ Participants received $60 at each baseline, 6-month, and 1-year assessment ($180) and additional $60 at each 6-month and 1-year assessment ($120) if they had provided ≥80% of their step counts and diet recalls during the previous 6 months.

### Outcome measures

The primary outcome, pre-specified and reported in the trial registration and methods paper,^15^ was the change from baseline to 1 year in the composite average, but not cumulative, *Z*-score of systolic blood pressure, LDL-C, fasting glucose, and per cent body fat; lower scores indicate better CVD risk profile. Each risk factor at baseline and follow-up was individually standardized to a sex-specific *Z*-score with a mean of 0 and standard deviation (SD) of 1 based on the formula ‘(observed value − mean)/SD’ for each participant using the baseline means and SDs from the entire sample. These four traditional CVD risk factors are often included in routine composite CVD risk algorithms to predict future CVD in clinical practice.^10,11,14,22^ Because the exact relative importance of each CVD risk factor was unclear in this specific population, we used the unweighted average of the four CVD risk factors, which is also common for a composite CVD risk score generation.^14^ However, to compare the relative contributions of each CVD risk factor to the composite CVD risk profile, we assessed the four individual CVD risk factors separately. We further assessed other emerging CVD risk factors (e.g. central blood pressure, waist circumference, cardiorespiratory fitness, and muscular strength) as secondary outcomes.

We assessed the outcomes at baseline, 6 months, and 1 year. After a minimum 12-h fast, morning assessments included a medical history questionnaire, peripheral and central blood pressure (the mean of three measurements with 2 min of rest between measurements) using an automated oscillometric device (SphygmoCor XECL), anthropometrics, body composition using a dual-energy X-ray absorptiometry (Horizon-W), and blood chemistry (Quest Diagnostics). Separate, non-fasted afternoon assessments included a physician-supervised maximal treadmill test for cardiorespiratory fitness and 1RM chest and leg press tests for muscular strength. The maximal treadmill test followed the Balke and Ware protocol,^23^ which is considered valid and safe for high-risk individuals, and then average heart rate and gas exchange variables including oxygen consumption (VO_2_), CO_2_, and respiratory exchange ratio (RER) were recorded every 30 s. Ratings of perceived exertion (RPE) using the Borg 6–20 scale were assessed every other minute and at volitional fatigue. Valid peak VO_2_ (VO_2peak_) values were determined using the ACSM criteria when participants reached RER ≥1.1, plateaued in VO_2_ or heart rate with increasing workload, or reported RPE >17.^16^ The 1RM tests were performed based on the National Strength and Conditioning Association guidelines.^24^ In brief, after three warm-up sets, participants performed a series of 1RM attempts with 2–4 min of rest between trials, and then the final maximal weight lifted successfully was considered the participant’s absolute 1RM. Detailed descriptions on maximal treadmill and 1RM tests were reported previously.^15^

### Statistical analyses

Using a linear mixed effects model at 5% significance, our pilot study indicated that 100 participants per group with 10% dropout would provide >90% power to detect a significant group-by-time interaction for the composite *Z*-score at 1 year (primary outcome), assuming *Z*-score reductions of 0.10, 0.10, and 0.19 in the resistance, aerobic, and combination groups, respectively, after adjusting for age, sex, and baseline composite *Z*-score value.^25^ The estimated power was also adequate (>90%) for the pre-specified comparisons (combination vs. resistance, combination vs. aerobic, resistance vs. control, and aerobic vs. control) with 90 completers per group allowing 10% dropout. We followed the intention-to-treat principle including all randomized participants in primary analyses. Missing values were handled by multivariate multiple imputation using Rubin’s method.^26^ We used linear mixed effects models with repeated measures including both baseline and follow-up data. When comparing the changes of the outcomes across groups, age, sex, and baseline value of each outcome were included as covariates. In three between-group comparisons (resistance vs. control, aerobic vs. control, and combination vs. control) for the primary and secondary outcomes, we further applied the Bonferroni correction to adjust for multiple comparisons. Per-protocol analysis included adherent participants with ≥80% exercise attendance who completed the study. We used SAS (9.4) and R (4.0.3) software, and all *P*-values were two sided.

## Results

### Participants and intervention fidelity

**Figure 1:**
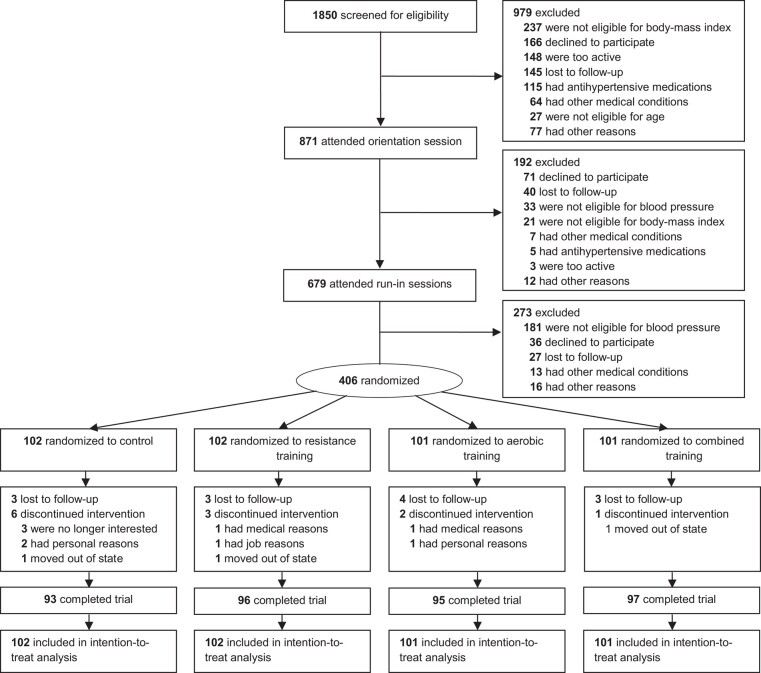
Participant screening, randomization, and follow-up. All these 406 participants were analyzed in the intention-to-treat analysis

Of 406 participants (53% women), 381 (94%) completed 1-year follow-up (*Figure 1*). Study participants were mostly white (80%) and well educated (*Table 1*). Exercise participants attended 82% of their prescribed exercise sessions over 1 year (84%, 77%, and 85% in the resistance, aerobic, and combination groups, respectively). The average exercise session was 61 min (59, 63, and 62 min in the resistance, aerobic, and combination groups, respectively). Participants exercised at 94% of their prescribed exercise intensity (95%, 97%, and 90% in the resistance, aerobic, and combination groups, respectively), calculated as an average performed %HRR divided by prescribed %HRR for the aerobic training and performed total weight divided by prescribed total weight for the resistance training following our earlier standardized procedures.^15,25^

**Table 1:** Baseline characteristics of the study participants

Characteristics	Control (*n* = 102)	Resistance (*n* = 102)	Aerobic (*n* = 101)	Combination (*n* = 101)	All participants (*n* = 406)
Age (years)	50 (10)	50 (10)	51 (10)	50 (10)	50 (10)
Female	55 (54%)	53 (52%)	53 (52%)	55 (54%)	216 (53%)
Body mass index (kg/m^2^)^a^	31.2 (4.8)	31.5 (5.2)	31.1 (4.8)	31.1 (5.0)	31.2 (4.9)
Race					
White	83 (81%)	81 (79%)	81 (80%)	81 (80%)	326 (80%)
Asian	8 (8%)	15 (15%)	10 (10%)	10 (10%)	43 (11%)
Black	3 (3%)	4 (4%)	2 (2%)	5 (5%)	14 (3%)
Other	8 (8%)	2 (2%)	8 (8%)	5 (5%)	23 (6%)
Hispanic ethnic group	7 (7%)	1 (1%)	3 (3%)	4 (4%)	15 (4%)
Education					
High school or less	12 (12%)	12 (12%)	7 (7%)	7 (7%)	38 (9%)
College degree	44 (44%)	45 (45%)	55 (55%)	53 (53%)	197 (49%)
Graduate school	46 (46%)	45 (45%)	39 (39%)	41 (41%)	171 (42%)
Marital status					
Single	10 (10%)	12 (12%)	8 (8%)	9 (9%)	39 (9%)
Married	83 (83%)	83 (83%)	85 (85%)	86 (86%)	337 (83%)
Divorced or separated	7 (7%)	6 (6%)	8 (8%)	6 (6%)	27 (7%)
Widowed	2 (2%)	1 (1%)	0 (0%)	0 (0%)	3 (1%)
Employment status					
Employed	83 (83%)	87 (87%)	85 (85%)	86 (86%)	341 (84%)
Retired	12 (12%)	5 (5%)	8 (8%)	7 (7%)	32 (8%)
Other	7 (7%)	10 (10%)	8 (8%)	8 (8%)	33 (8%)
Medical conditions					
Diabetes mellitus	1 (1%)	2 (2%)	1 (1%)	4 (4%)	8 (2%)
Hypercholesterolaemia	25 (25%)	19 (19%)	29 (29%)	17 (17%)	90 (22%)
Parental CVD	34 (34%)	35 (35%)	38 (38%)	31 (31%)	138 (34%)
Physical activity (min/week)^b^					
Moderate aerobic activity	95 (110)	87 (145)	93 (86)	92 (107)	92 (114)
Vigorous aerobic activity	13 (24)	20 (32)	22 (34)	18 (32)	18 (31)
Muscle-strengthening activity	16 (43)	10 (20)	15 (29)	12 (31)	13 (32)

Data are mean (SD) or *n* (%). No significant group differences were found in all baseline characteristics.

CVD, cardiovascular disease.

^a^Calculated as weight in kilograms divided by height in metres squared.

^b^Examples of moderate aerobic activities include brisk walking, house cleaning, washing car, or general gardening; examples of vigorous aerobic activities include running, singles tennis, or basketball; and examples of muscle-strengthening activities include weight training, push-ups, carrying heavy loads, or heavy gardening.

### Primary outcome

**Figure 2:**
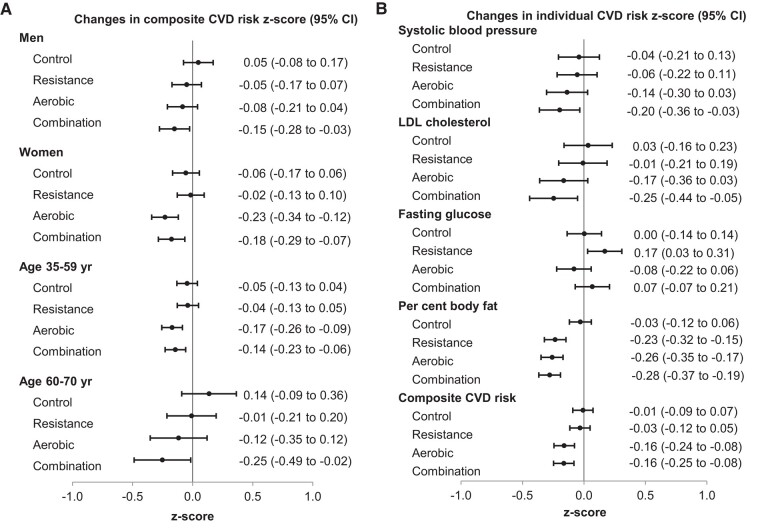
Changes in the cardiovascular disease risk factors from baseline to 1 year. Results are from the linear mixed effects models with repeated measures in the intention-to-treat analyses including all 406 randomized participants. The figure shows mean changes in the standardized *Z*-scores of the composite cardiovascular disease risk factors by sex and age groups (*A*) and mean changes in the standardized *Z*-scores of the individual and composite cardiovascular disease risk factors (*B*). *P*-values for interaction by sex were .22, .70, .09, and .78 in the control, resistance, aerobic, and combination groups, respectively. *P*-values for interaction by the age groups were .14, .79, .67, and .40 in the control, resistance, aerobic, and combination groups, respectively. *Z*-score values below 0 indicate favourable changes in cardiovascular disease risk factors. Whiskers indicate 95% confidence intervals. CVD, cardiovascular disease; LDL, CI, confidence interval

There were decreases in the composite CVD risk *Z*-score [95% confidence interval (CI)] at 1 year in the aerobic vs. control [difference from control, −0.15 (−0.27 to −0.04); *P* = .01] and combination vs. control [difference, −0.16 (−0.27 to −0.04); *P* = .009] in the intention-to-treat analysis (*Table 2*). There was however no significant difference in the resistance vs. control [difference, −0.02 (−0.14 to 0.09); *P* = .69]. Between the three time-matched exercise groups, we found greater reductions in the composite *Z*-score at 1 year in the aerobic vs. resistance [difference, −0.13 (−0.25 to −0.01); *P* = .03] and combination vs. resistance [difference, −0.13 (−0.25 to −0.02); *P* = .03] but no difference in the combination vs. aerobic [difference, 0.00 (−0.12 to 0.11); *P* = .96; *Table 3*]. The magnitude of the reductions in the composite *Z*-score from baseline to 1 year within the aerobic or combination group was equal (both −0.16). We generally found similar patterns in men (*n* = 190), women (*n* = 216), younger (35–59 years; *n* = 322), and older (60–70 years; *n* = 84) participants (*Figure 2A*), although the variability in the response to the exercise appears to be larger in the older participants, partly due to the smaller sample size. There was no significant interaction by sex or the age groups.

**Table 2:** Effect of resistance, aerobic, and combined exercise on primary and secondary outcomes at 1 year in intention-to-treat analyses

	Control (*n* = 102)	Resistance (*n* = 102)	Aerobic (*n* = 101)	Combination (*n* = 101)	Between-group difference (95% CI)^a^		
					Resistance vs. control	Aerobic vs. control	Combination vs. control
Primary outcome							
Composite CVD risk *Z*-score^b^							
Baseline mean (SD)	0.00 (0.57)	0.02 (0.55)	−0.01 (0.56)	0.00 (0.55)			
Change at 1 year (95% CI)	−0.01 (−0.09 to 0.07)	−0.03 (−0.12 to 0.05)	−0.16 (−0.24 to −0.08)	−0.16 (−0.25 to −0.08)	−0.02 (−0.14 to 0.09)	−0.15 (−0.27 to −0.04)	−0.16 (−0.27 to −0.04)
*P*-value	.85	.44	<.001	<.001	.69	.01	.009
Secondary outcomes							
Blood pressure, mmHg							
Peripheral systolic							
Baseline mean (SD)	127.0 (11.3)	128.3 (10.2)	127.6 (10.7)	128.3 (11.4)			
Change at 1 year (95% CI)	−0.5 (−2.3 to 1.4)	−0.6 (−2.3 to 1.2)	−1.5 (−3.3 to 0.3)	−2.2 (−3.9 to −0.4)	−0.1 (−2.7 to 2.4)	−1.0 (−3.6 to 1.5)	−1.7 (−4.3 to 0.9)
*P*-value	.62	.52	.11	.02	.93	.43	.19
Peripheral diastolic							
Baseline mean (SD)	79.5 (8.0)	79.8 (7.2)	80.0 (7.4)	79.4 (7.2)			
Change at 1 year (95% CI)	−0.8 (−2.1 to 0.4)	−0.7 (−1.9 to 0.5)	−1.5 (−2.7 to −0.2)	−0.8 (−2.0 to 0.5)	0.1 (−1.6 to 1.9)	−0.6 (−2.4 to 1.1)	0.1 (−1.7 to 1.8)
*P*-value	.20	.27	.02	.23	.88	.48	.94
Central systolic							
Baseline mean (SD)	117.1 (10.8)	118.5 (9.5)	118.3 (10.2)	118.4 (10.3)			
Change at 1 year (95% CI)	−0.2 (−1.8 to 1.4)	−0.4 (−2.0 to 1.2)	−1.3 (−3.0 to 0.3)	−1.5 (−3.1 to 0.1)	−0.2 (−2.5 to 2.1)	−1.1 (−3.5 to 1.2)	−1.3 (−3.7 to 1.0)
*P*-value	.81	.61	.11	.06	.85	.33	.26
Central diastolic							
Baseline mean (SD)	80.5 (7.9)	80.6 (7.3)	81.0 (7.4)	80.3 (7.5)			
Change at 1 year (95% CI)	−1.0 (−2.3 to 0.3)	−0.6 (−1.9 to 0.6)	−1.6 (−2.9 to −0.4)	−0.7 (−1.9 to 0.5)	0.4 (−1.4 to 2.2)	−0.6 (−2.5 to 1.2)	0.3 (−1.5 to 2.1)
*P*-value	.13	.32	.01	.27	.69	.51	.74
Cholesterol levels, mg/dL							
LDL cholesterol							
Baseline mean (SD)	123.2 (30.9)	120.3 (28.7)	122.5 (39.8)	124.4 (27.8)			
Change at 1 year (95% CI)	0.8 (−5.4 to 7.0)	−0.3 (−6.5 to 5.9)	−5.5 (−11.7 to 0.7)	−7.8 (−13.9 to −1.6)	−1.1 (−9.9 to 7.7)	−6.3 (−15.0 to 2.4)	−8.6 (−17.3 to 0.1)
*P*-value	.79	.93	.08	.01	.81	.16	.05
HDL cholesterol							
Baseline mean (SD)	48.8 (14.8)	48.0 (15.2)	46.7 (12.2)	48.0 (14.2)			
Change at 1 year (95% CI)	−0.8 (−2.1 to 0.5)	1.1 (−0.2 to 2.4)	1.2 (−0.1 to 2.5)	1.5 (0.2–2.8)	1.9 (0.1–3.7)	2.0 (0.2–3.9)	2.3 (0.5–4.1)
*P*-value	.23	.09	.06	.02	.04	.03	.01
Fasting glucose, mg/dL							
Baseline mean (SD)	95.8 (11.3)	95.8 (9.9)	95.9 (12.8)	96.2 (12.5)			
Change at 1 year (95% CI)	0.0 (−1.6 to 1.6)	1.9 (0.3–3.5)	−1.1 (−2.8 to 0.5)	0.6 (−1.1 to 2.2)	1.9 (−0.4 to 4.2)	−1.1 (−3.4 to 1.2)	0.6 (−1.7 to 2.9)
*P*-value	.99	.02	.17	.48	.10	.33	.61
Body composition							
Per cent body fat, %							
Baseline mean (SD)	38.6 (7.0)	38.5 (7.4)	38.2 (7.2)	37.9 (6.8)			
Change at 1 year (95% CI)	−0.1 (−0.5 to 0.2)	−1.0 (−1.3 to −0.6)	−1.1 (−1.5 to −0.7)	−1.2 (−1.5 to −0.8)	−0.9 (−1.4 to −0.3)	−1.0 (−1.5 to −0.4)	−1.0 (−1.6 to −0.5)
*P*-value	.51	<.001	<.001	<.001	.001	<.001	<.001
Body weight, kg							
Baseline mean (SD)	91.4 (17.5)	91.7 (18.1)	90.4 (17.6)	90.7 (17.8)			
Change at 1 year (95% CI)	−0.1 (−1.0 to 0.7)	0.6 (−0.2 to 1.5)	−1.4 (−2.3 to −0.6)	−1.3 (−2.2 to −0.5)	0.8 (−0.4 to 2.0)	−1.3 (−2.5 to −0.1)	−1.2 (−2.4 to 0.0)
*P*-value	.74	.15	.001	.003	.21	.04	.06
Waist circumference, cm							
Baseline mean (SD)	102.6 (12.3)	103.3 (12.7)	103.2 (12.4)	102.3 (12.7)			
Change at 1 year (95% CI)	−0.7 (−1.9 to 0.6)	−0.8 (−2.0 to 0.4)	−2.6 (−3.8 to −1.4)	−2.9 (−4.1 to −1.7)	−0.1 (−1.9 to 1.6)	−1.9 (−3.7 to −0.2)	−2.2 (−3.9 to −0.5)
*P*-value	.29	.18	<.001	<.001	.87	.03	.01
Lean body mass, kg							
Baseline mean (SD)	52.4 (11.1)	52.5 (11.1)	52.2 (12.0)	52.5 (11.2)			
Change at 1 year (95% CI)	0.0 (−0.5 to 0.4)	1.2 (0.8–1.7)	0.0 (−0.4 to 0.5)	0.3 (−0.2 to 0.7)	1.2 (0.6–1.9)	0.0 (−0.6 to 0.7)	0.3 (−0.4 to 0.9)
*P*-value	.92	<.001	.91	.27	<.001	.88	.39
Physical fitness							
VO_2peak_, mL/kg/min^c^							
Baseline mean (SD)	26.3 (6.6)	25.5 (6.0)	26.1 (6.2)	26.5 (6.7)			
Change at 1 year (95% CI)	−1.0 (−1.9 to −0.1)	0.3 (−0.6 to 1.2)	2.5 (1.6–3.5)	1.7 (0.8–2.6)	1.3 (0.01–2.5)	3.5 (2.2–4.8)	2.7 (1.5–4.0)
*P*-value	.03	.54	<.001	<.001	.048	<.001	<.001
1RM chest press, kg^d^							
Baseline mean (SD)	52.8 (22.2)	57.1 (24.2)	53.2 (23.8)	54.6 (22.7)			
Change at 1 year (95% CI)	−0.4 (−2.0 to 1.2)	10.9 (9.3–12.5)	−1.2 (−2.8 to 0.5)	6.8 (5.3–8.4)	11.3 (9.0–13.6)	−0.7 (−3.0 to 1.5)	7.3 (5.0–9.5)
*P*-value	.62	<.001	.16	<.001	<.001	.52	<.001
1RM leg press, kg^d^							
Baseline mean (SD)	122.0 (49.9)	126.5 (49.0)	125.7 (53.5)	125.7 (51.5)			
Change at 1 year (95% CI)	5.1 (−0.5 to 10.6)	25.4 (19.8–30.9)	3.2 (−2.5 to 8.8)	14.9 (9.4–20.4)	20.3 (12.4–28.2)	−1.9 (−9.9 to 6.0)	9.8 (2.1–17.5)
*P*-value	.07	<.001	.28	<.001	<.001	.63	.01

Baseline means (SDs) are the unadjusted values and the changes (95% CIs) from baseline to 1 year are the least squares adjusted values including age, sex, and baseline value of each outcome as covariates in the intention-to-treat analyses including all 406 randomized participants.

^a^ *P*-values in between-group differences were calculated using linear mixed effects models with repeated measures including age, sex, and baseline value of each outcome as covariates in the intention-to-treat analyses including all 406 randomized participants.

^b^Composite CVD risk *Z*-score is the mean of the sex-specific *Z*-scores of four established CVD risk factors: resting systolic blood pressure, LDL cholesterol, fasting glucose, and per cent body fat, with lower scores indicating lower overall CVD risk profile.

^c^Peak oxygen consumption (VO_2peak_, measured as millilitres per kilogram of body weight per minute) was assessed during a maximal graded treadmill test using the Balke and Ware protocol.

^d^One-repetition maximum (1RM) is the maximum weight a participant can lift in one attempt in the chest press or leg press.

**Table 3:** Comparisons of resistance, aerobic, and combined exercise on primary outcome at 1 year in intention-to-treat analyses

Primary outcome	Resistance (*n* = 102)	Aerobic (*n* = 101)	Combination (*n* = 101)	Between-group difference (95% CI)^a^		
				Aerobic vs. resistance	Combination vs. resistance	Combination vs. aerobic
Composite CVD risk *Z*-score**^b^**						
Baseline mean (SD)	0.02 (0.55)	−0.01 (0.56)	0.00 (0.55)			
Change at 1 year (95% CI)	−0.03 (−0.12 to 0.05)	−0.16 (−0.24 to −0.08)	−0.16 (−0.25 to −0.08)	−0.13 (−0.25 to −0.01)	−0.13 (−0.25 to −0.02)	0.00 (−0.12 to 0.11)
*P*-value	.44	<.001	<.001	.03	.03	.96

Baseline means (SDs) are the unadjusted values and the changes (95% CIs) from baseline to 1 year are the least squares adjusted values including age, sex, and baseline value of the outcome as covariates in the intention-to-treat analyses including all 406 randomized participants.

^a^ *P*-values in between-group differences were calculated using linear mixed effects models with repeated measures including age, sex, and baseline value of the outcome as covariates in the intention-to-treat analyses including all 406 randomized participants.

^b^Composite CVD risk *Z*-score is the mean of the sex-specific *Z*-scores of four established CVD risk factors: resting systolic blood pressure, LDL cholesterol, fasting glucose, and per cent body fat, with lower scores indicating lower overall CVD risk profile.

When the Bonferroni correction was additionally applied, the results were the same suggesting that both aerobic (*P* = .03) and combination (*P* = .03), but not resistance (*P* > .99), groups showed reductions in the composite *Z*-score compared with the change in the control group at 1 year. In sensitivity analyses using the complete data after excluding missing data, both aerobic (*P* = .01) and combination (*P* = .006) groups, but not the resistance (*P* = .64) group, also showed larger decreases in the composite *Z*-score compared with the control group at 1 year.

### Secondary outcomes

Regarding the four individual CVD risk factors included in the composite *Z*-score, per cent body fat (95% CI) decreased at 1 year in the resistance vs. control [difference, −0.9% (−1.4 to −0.3); *P* = .001], aerobic vs. control [difference, −1.0% (−1.5 to −0.4); *P* < .001], and combination vs. control [difference, −1.0% (−1.6 to −0.5); *P* < .001; *Table 2*]. However, changes in systolic blood pressure, LDL-C, and fasting glucose in the exercise groups were not statistically different compared with the control. Regarding other CVD risk factors, we found increases in HDL cholesterol (HDL-C; 95% CI) in the resistance vs. control [difference, 1.9 mg/dL (0.1–3.7); *P* = .04], aerobic vs. control [difference, 2.0 mg/dL (0.2–3.9); *P* = .03], and combination vs. control [difference, 2.3 mg/dL (0.5–4.1); *P* = .01]; decreases in body weight in the aerobic vs. control [difference, −1.3 kg (−2.5 to −0.1); *P* = .04]; decreases in waist circumference in the aerobic vs. control [difference, −1.9 cm (−3.7 to −0.2); *P* = .03] and combination vs. control [difference, −2.2 cm (−3.9 to −0.5); *P* = .01]; and increases in lean body mass in the resistance vs. control [difference, 1.2 kg (0.6–1.9); *P* < .001] at 1 year (*Table 2*). However, no significant differences were found between the exercise groups and the control in other CVD risk factors.

This study further compared the standardized Z-score in all four individual CVD risk factors (*Figure 2B*) and found larger *Z*-score reductions in per cent body fat followed by LDL-C and systolic blood pressure in the aerobic and combination groups, as the predominant contributor to the change of the composite CVD risk profile at 1 year.

**Figure 3:**
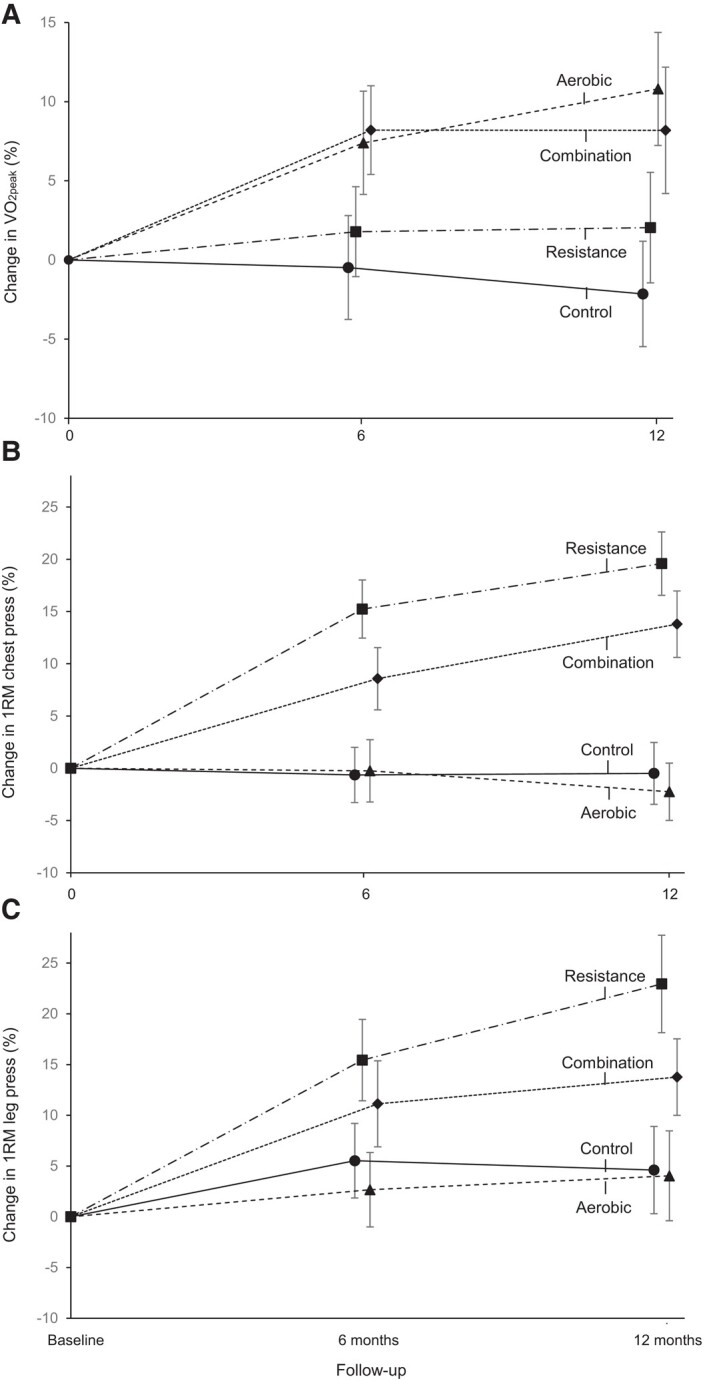
Mean per cent changes in cardiorespiratory fitness and muscular strength from baseline to 6 and 12 months. Results are from the linear mixed effects models with repeated measures in the intention-to-treat analyses including all 406 randomized participants. The figure shows cardiorespiratory fitness (*A*) measured in the peak oxygen consumption, one-repetition maximum chest press (*B*), and one-repetition maximum leg press (*C*) measured at baseline, 6 months, and 12 months. Error bars represent 95% confidence intervals. 1RM, one-repetition maximum (the maximum weight a participant can lift in one attempt); VO_2peak_, peak oxygen consumption

Cardiorespiratory fitness (95% CI) increased in the resistance vs. control [difference, 1.3 mL/kg/min (0.01–2.5); *P* = .048], aerobic vs. control [difference, 3.5 mL/kg/min (2.2–4.8); *P* < .001], and combination vs. control [difference, 2.7 mL/kg/min (1.5–4.0); *P* < .001] at 1 year (*Table 2*). We found no difference between the aerobic and combination groups (*P* = .24), but both aerobic (*P* = .001) and combination (*P* = .02) groups improved cardiorespiratory fitness more than the resistance group. We found similar results using the absolute value of VO_2peak_ in litres per minute in additional analyses. Both 1RM chest and leg press (95% CI) increased in the resistance vs. control [differences, 11.3 kg (9.0–13.6); *P* < .001 and 20.3 kg (12.4–28.2); *P* < .001, respectively] and combination vs. control [differences, 7.3 kg (5.0–9.5); *P* < .001 and 9.8 kg (2.1–17.5); *P* = .01, respectively] at 1 year. The resistance group improved chest and leg press more than the combination group (both *P* < .01). During the 1-year intervention period, cardiorespiratory fitness in the aerobic group and muscular strength in the resistance group continued to increase from baseline through 6 months to 1 year (*Figure 3*).

When the Bonferroni correction was additionally applied, compared with the change in the control group, HDL-C increased only in the combination (*P* = .03), but not in the resistance (*P* = .12) or aerobic (*P* = .09), group; waist circumference decreased only in the combination (*P* = .03), but not in the aerobic (*P* = .09), group; and body weight no longer decreased in the aerobic (*P* = .12) group. However, compared with the change in the control group, per cent body fat still decreased in all resistance (*P* = .003), aerobic (*P* < .001), and combination (*P* < .001) groups; lean body mass increased in the resistance (*P* < .001) group; cardiorespiratory fitness increased in both aerobic (*P* < .001) and combination (*P* < .001), but not in the resistance (*P* = .14), groups; and 1RM chest and leg press increased in both resistance (both *P* < .001) and combination (*P* < .001 and *P* = .03, respectively) groups. No significant changes were found on other outcomes.

In additional analyses, we estimated the changes in 10-year coronary heart disease (CHD) risk score between baseline and 1 year using the Framingham risk score according to age, diabetes, smoking, blood pressure, LDL-C, and HDL-C, developed in a 30–74-year-old, predominantly white population,^22^ which is similar to ours. The risk of developing CHD over 10 years decreased by −1.1% in the combination group (from 7.2% at baseline to 6.1% at 1 year; *P* < .001), although no significant changes were found in the aerobic (−0.5% from 6.5% to 6.0%; *P* = .10) and resistance (0.1% from 6.3% to 6.4%; *P* = .76) groups. Compared with the control group, the decreased CHD risk in the combination group was larger (*P* = .03), but no differences were found in the aerobic and resistance groups. However, the Framingham risk score may underestimate the overall cardiovascular benefits of exercise because the equation does not include per cent body fat that improved in all exercise groups in this study of adults with overweight or obesity.

In sensitivity analyses, we added HDL-C, which is included in the Framingham risk score, into the original composite *Z*-score, and found slightly improved change in the composite *Z*-score [95% CI; −0.04 (−0.12 to 0.03)] at 1 year in the resistance group, yet not statistically different from the change in the control group (*P* = .37), although the improvements in the aerobic and combination groups remained significant. When we further added four emerging CVD risk factors [central systolic blood pressure, waist circumference, cardiorespiratory fitness, and average muscular strength (mean 1RM chest and leg press)], we found that the extended composite *Z*-score (95% CI) was improved in all resistance [−0.13 (−0.19 to −0.07)], aerobic [−0.18 (−0.24 to −0.12)], and combination [−0.22 (−0.28 to −0.16)] groups than in the control [0.00 (−0.06 to 0.06)] group at 1 year (all *P* < .01 compared with the control). We used reverse score (multiplied by −1) for HDL-C, cardiorespiratory fitness, and muscular strength, which have inverse relationships with CVD.

**Figure 4:**
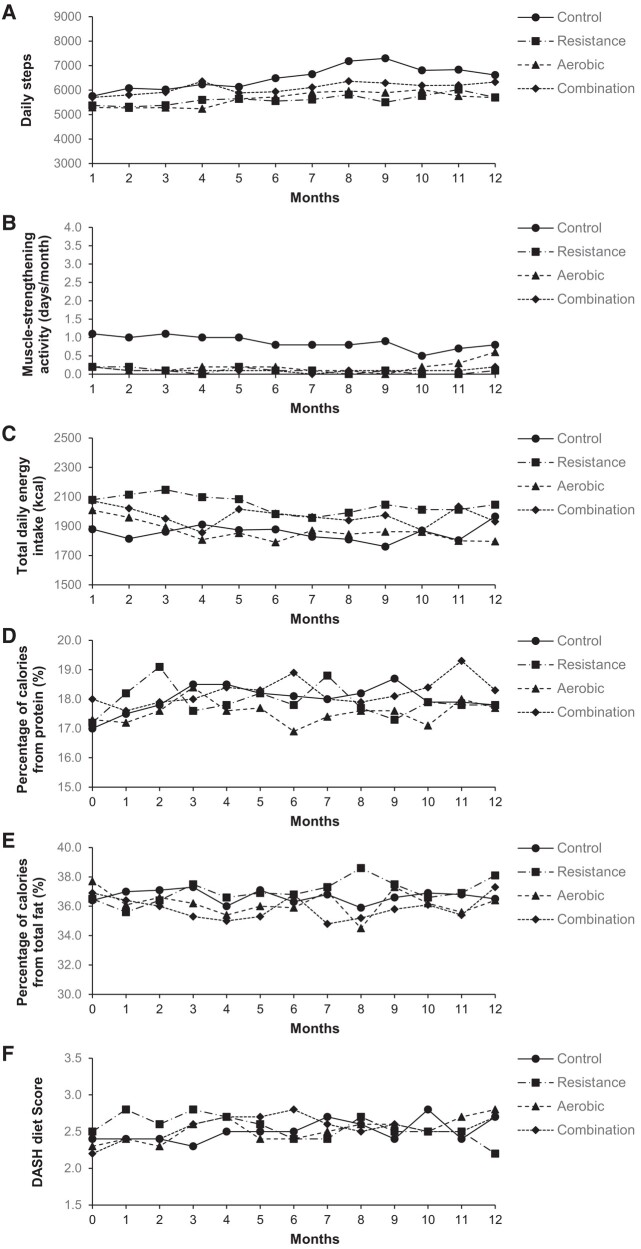
Mean changes in lifestyle physical activity and diet during 12 months. The figure shows the mean changes in daily steps (*A*), muscle-strengthening activity (*B*), total daily energy intake (*C*), percentage of calories from protein (*D*), percentage of calories from total fat (*E*), and Dietary Approaches to Stop Hypertension diet score (*F*). Average numbers of participants across groups from Month 1 to Month 12 were 99, 95, 93, 92, 89, 87, 84, 80, 78, 76, 74, and 73, respectively, for physical activity (daily steps and muscle-strengthening activity) and 95, 88, 88, 83, 81, 79, 76, 74, 72, 68, 66, and 46, respectively, for diet (total daily energy intake, percentage of calories from protein, percentage of calories from total fat, and Dietary Approaches to Stop Hypertension diet score). *P*-values for group-by-time interactions are as follows: *P* = .37 for daily steps, *P* = .97 for muscle-strengthening activity, *P* = .67 for total daily energy intake, *P* = .42 for percentage of calories from protein, *P* = .43 for percentage of calories from total fat, and *P* = .53 for Dietary Approaches to Stop Hypertension diet score. DASH, Dietary Approaches to Stop Hypertension [ranges from 0 (low DASH accordance) to 9 (high DASH accordance)].

At 6 months, only the combination group had a decrease in the composite *Z*-score compared with the control group [difference, −0.13 (−0.25 to −0.02); *P* = .03], although the changes in the composite *Z*-score within all groups, including the control group, appeared to be slightly larger at 6 months than the changes at 1 year (see Supplementary data online, *Table S1* and *Figure S1*). This may be, in part, related to the relatively higher exercise adherence during the first 6 months than the second 6 months (average exercise attendance rate in all exercise groups was 86% at first 6 months vs. 75% at second 6 months), which is typical in long-term exercise trials.^12,17,27^ Improvements in CVD risk factors, including the composite *Z*-score, in the exercise groups tended to be larger in the per-protocol analyses (*n* = 313, 77%) with a higher (≥80%) exercise attendance rate (see Supplementary data online, *Table S2*). We found no significant differences in changes in daily steps, muscle-strengthening activity, total energy intake, the percentage of calories from macronutrients, and DASH diet accordance score^28^ among the four groups over 1 year (*Figure 4*). However, despite the non-significant difference, it is plausible that there might have been some influence on the results due to more daily steps and muscle-strengthening activity in the control group than the other groups.

### Adverse events

Four participants (1%) had study-related, non-serious adverse events (e.g. shoulder pain) with no significant differences among groups (see Supplementary data online, *Table S3*).

## Discussion

Aerobic exercise alone or combined resistance plus aerobic exercise, but not resistance exercise alone, improved composite CVD risk profile relative to no-exercise control in adults with overweight or obesity (*Structured Graphical Abstract*). The combination group that did half (30 min/session) of each resistance and aerobic exercise improved composite CVD risk profile similar to the full (60 min/session) aerobic exercise alone. This supports the current physical activity guidelines recommending both resistance and aerobic exercise by the USA, WHO,^3,4^ and European Society of Cardiology (specifically for individuals with obesity).^5^ Our finding further provides evidence-based data that replacing half of aerobic exercise with resistance exercise, without extra exercise time, may be an effective option to improve CVD risk profile in adults with overweight or obesity.

There are limited prior trials directly comparing the effects of resistance, aerobic, and combined exercise training on composite CVD risk profiles. However, regarding the four individual CVD risk factors included in the composite CVD risk profile, meta-analyses suggested that aerobic or combined exercise is generally superior to resistance exercise on blood pressure,^29^ body fat,^30^ glucose metabolism, and lipid profile,^31,32^ although these data were mostly from small (*n* < 30 per group) and short (2–6 months) exercise interventions. Observational studies also suggest that combined exercise provides somewhat larger (although generally not statistically different compared with either resistance or aerobic exercise alone) risk reductions in developing clinical endpoints such as metabolic syndrome,^33^ hypercholesterolaemia,^34^ obesity,^35^ and CVD morbidity and mortality.^36–38^ These data further suggest that shorter durations of aerobic or resistance exercise (even <1 h/week) may be sufficient to provide substantial CVD benefits, with diminishing returns with increasing exercise time, which may explain the significant improvement in the composite CVD risk profile with half of aerobic and resistance exercise in the combination group. Considering the inherent limitations of observational studies such as measurement errors (e.g. over-reported exercise), potential reverse causation, and residual confounding factors, the current study provides experimental data on the direction and magnitude of the effects of different types of exercise on CVD risk profile in a general adult population with overweight or obesity. We found however that lean body mass was improved at 1 year only in the resistance group compared with the control group, which is a key to maintain a healthy weight, thus could be considered to preserve muscle mass in weight loss programmes that induce muscle loss in individuals with obesity.^13^ Resistance exercise may also be a more accessible type of exercise for less mobile individuals with overweight or obesity who may find aerobic exercise (e.g. running) less tolerable due to their heavy weight.

Regarding the primary outcome, the reductions of −0.16 in the composite *Z*-score in both aerobic and combination groups at 1 year, which were larger than the change in the control group, are meaningful. The Healthy Ageing through Internet Counselling in the Elderly (HATICE) trial observed a reduction of −0.09 in their composite average *Z*-score, derived from systolic blood pressure, LDL-C, and BMI after an 18-month healthy lifestyle intervention including physical activity counselling.^39^ This composite *Z*-score reduction of −0.09 was associated with hazard ratios (95% CIs) of 0.86 (0.52–1.43) and 0.30 (0.10–0.93) for incident total CVD and stroke risk, respectively, compared to the control group in 2724 older adults, although 95% CIs were wide, and the hazard ratios were likely based on limited numbers of events. When we used the same three components (systolic blood pressure, LDL-C, and BMI) of the composite *Z*-score, we observed composite *Z*-score reductions of −0.13 and −0.19 at 1 year in the aerobic and combination groups, respectively.

Several exercise trials combined with weight loss reported larger improvements in CVD risk factors.^21,40,41^ However, our study was designed to induce no or negligible weight loss to investigate the independent effects of exercise on CVD risk factors. With <−1.5 kg lost at 1 year, the current study found improved CVD risk profile in the aerobic and combination groups, which was consistent after further adjustment for body weight change. However, the findings of −1% body fat reduction, although not large, in all exercise groups may be noteworthy because every −1% body fat reduction is associated with −3%, −4%, and −8% lower risks of developing CVD risk factors of hypertension, hypercholesterolaemia, and metabolic syndrome, respectively, in our early study.^42^ Considering the growing global epidemic of obesity and its strong associations with CVD progression,^6^ the improvements in per cent body fat and CVD risk profile through exercise with minimal weight loss may be important given that weight loss is challenging, especially in individuals with overweight or obesity.

The magnitudes of the changes in the individual CVD risk factors in this study are mostly modest, which is possibly related to the relatively healthy participants with mildly elevated average baseline systolic blood pressure (128 mmHg) without taking antihypertensive medications as well as normal average LDL-C (123 mg/dL) and fasting glucose (96 mg/dL) who might have had limited room for improvement. Earlier studies also reported that the improvements in CVD risk factors were generally larger in patient populations,^43^ including greater metabolic benefits in people living with diabetes,^12,17^ which may explain the lack of significant changes in fasting glucose in our sample mostly without diabetes (98%). We also examined diabetes and hypercholesterolaemia medications. At baseline, 45 (11%) participants (resistance = 9, aerobic = 15, combination = 8, and control = 13) used diabetes (*n* = 5), hypercholesterolaemia (*n* = 42), or both (*n* = 2) medications. Of those 45 participants, 12 (27%) (resistance = 3, aerobic = 5, combination = 2, and control = 2) either stopped or reduced their medications at 1 year. However, the results on the primary outcome remained the same after further adjustment for the changes in the medications in additional analyses.

Another possible explanation of the modest changes in the CVD risk factors may be related to the fact that study participants were not completely inactive at baseline (e.g. with 92 min/week of moderate aerobic activity, shown in *Table 1*), although they reported not meeting the exercise guidelines. Thus, the effects of exercise could be somewhat blunted in these participants. However, our modest results align with other long-term (≥6 months) non-weight loss exercise trials (mostly aerobic exercise) in populations with similar BMIs (29–35 kg/m^2^) that have also shown small changes in individual CVD risk factors, reporting reductions of −1 to −3 mmHg in systolic blood pressure, 0 to −6 mg/dL in LDL-C, −1 to −2 mg/dL in fasting glucose, and −1 to −2% in per cent body fat.^12,20,27,44^

This study underscores important findings on cardiorespiratory fitness and muscular strength by different types of exercise in relation to CVD prevention. The mean increases of 1.0 and 0.8 METs, metabolic equivalents [one MET is equal to 3.5 mL/kg/min of oxygen consumption (VO_2_)], in cardiorespiratory fitness in the aerobic and combination groups, respectively, compared with the control group may have clinical importance since every 1 MET increase over time has been associated with 19% lower CVD mortality risk in our early study,^45^ supported by a meta-analysis.^46^ Regarding the muscular strength improvements in the resistance and combination groups, a large prospective study found that every 5 kg reduction in grip strength was associated with 7%, 9%, and 17% increased risk of myocardial infarction, stroke, and CVD mortality, respectively.^47^ Combined resistance plus aerobic exercise could be considered to increase both cardiorespiratory fitness and muscular strength that are also important to attenuate the harmful effects of obesity on CVD prevention and longevity.^35,45,47^

### Strengths and limitations

This is one of the longest and largest supervised exercise trials with 406 participants, 94% retention, and 82% average exercise attendance. This study extensively monitored physical activity and diet outside the lab and found no significant changes throughout 1 year across the groups, which may minimize possible confounding effects because exercise study participants are motivated to be active and follow healthy diet that may have provided larger benefits on CVD risk factors in earlier studies without outside activity and diet monitoring throughout their intervention. We found that cardiorespiratory fitness and muscular strength, potential markers of successful delivery of the aerobic and resistance training, respectively, improved continuously over 1 year, which may support the robustness of the findings. We used a commercially available computer-controlled exercise training system, so the interventions could be prescribed and monitored precisely, yet the programmatic aspects of this study could be implemented broadly (e.g. health club settings).

This study also has important limitations to consider. First, most participants were white and well educated, so the results cannot be generalized to more diverse populations. Second, this single-centre supervised exercise trial was designed as an efficacy study; thus, the effects of exercise outside the supervised lab in real world are likely to be different. Third, whether an improved composite CVD risk score by exercise training lowers the risk of developing CVD (e.g. heart attack and stroke) in a long-term follow-up is unknown and warrants further investigation. Fourth, this study used time (60 min/session) to equalize the exercise delivery between groups for more direct public health implications; thus, the findings may be different from other studies that used energy expenditure to equalize the volume of exercise between exercise groups. Fifth, like other lifestyle interventions, participants knew what group they were in although we attempted to minimize this potential limitation by blinding outcome assessment staff to group allocations and excluding intervention staff from assessments. Sixth, we excluded active individuals meeting the guidelines using self-report at baseline; thus, some may have underreported their activity to participate in the study, or those who over-reported their activity were unnecessarily excluded. Therefore, future studies should consider objectively measured physical activity, if used as an eligibility criterion, to reduce the possible confounding effects of baseline activity. Seventh, diet was self-reported via 24-h recall; thus, possible measurement errors and recall bias should be considered in the outcome variables (e.g. lipid profile and body weight). A pedometer would also not capture all types of physical activities outside of exercise sessions (e.g. cycling). Lastly, the average exercise attendance rate at 1 year was lower in the aerobic (77%) compared with resistance (84%, *P* = .04) or combination (85%, *P* = .02) group, similar to an earlier study.^17^ However, in the per-protocol analysis with a minimum 80% exercise attendance rate, the aerobic group showed a slightly larger reduction in the composite *Z*-score (−0.22) than the combination group (−0.19) at 1 year. Therefore, it is possible that aerobic exercise may be more efficacious to improve CVD risk profile if performed at a sufficient dose, but combined exercise may be more effective in the long run, considering higher exercise adherence.

## Conclusions

To improve CVD risk profile in adults with overweight or obesity, this study suggests that aerobic exercise needs to be included. However, replacing half of aerobic exercise with resistance exercise may be an effective alternative that provides similar improvements in CVD risk profile with the additional benefit of increasing muscular strength that aerobic exercise alone does not provide. These findings may help develop clinical and public health practices and recommendations for the ∼2 billion adults with overweight or obesity worldwide who are at increased risk of CVD.^6^ However, caution is needed in interpreting the results from this single-centre efficacy trial, and large effectiveness studies are required.

## Supplementary Material

ehad827_Supplementary_Data

## Data Availability

Deidentified data will be made available to researchers after approval of a proposal by the trial investigator team. Proposals for data use, with justification, should be submitted to the corresponding author (dclee@iastate.edu) for consideration.

## Pre-registered Clinical Trial Number

The pre-registered clinical trial number is NCT03069092.
