# Supplementary material for: Aerobic, resistance, or combined exercise training and cardiovascular risk profile in overweight or obese adults: the CardioRACE trial
Source: Eur Heart J. 2024 Jan 17;45(13):1127–42. doi: 10.1093/eurheartj/ehad827 (PMC10984570; doi:10.1093/eurheartj/ehad827)
Supplement: ehad827_Supplementary_Data [file ehad827_supplementary_data.docx]

**Supplementary Materials**

**Table of Contents**

- **Supplementary Table S1:** Effect of resistance, aerobic, and combined exercise on primary and secondary outcomes at 6 months in intention-to-treat analyses
- **Supplementary Table S2:** Effect of resistance, aerobic, and combined exercise on primary and secondary outcomes at 6 months and 1 year in per-protocol analyses
- **Supplementary Table S3:** Adverse events
- **Supplementary Figure S1:** Changes in the CVD risk factors from baseline to 6 months

| **Supplementary Table S1:** Effect of resistance, aerobic, and combined exercise on primary and secondary outcomes at 6 months in intention-to-treat analyses | | | | | | | |
| --- | --- | --- | --- | --- | --- | --- | --- |
| **Outcome** | **Control**  **(n = 102)** | **Resistance**  **(n = 102)** | **Aerobic**  **(n = 101)** | **Combination**  **(n = 101)** | **Between-group difference (95% CI)*** | | |
|  |  |  |  |  | **Resistance**  **vs Control** | **Aerobic**  **vs Control** | **Combination**  **vs Control** |
| **Primary outcome** |  |  |  |  |  |  |  |
| **Composite CVD risk z-score**† |  |  |  |  |  |  |  |
| Baseline mean (SD) | 0.00 (0.57) | 0.02 (0.55) | -0.01 (0.56) | 0.00 (0.55) |  |  |  |
| Change at 6 months (95% CI) | -0.08  (-0.16 to 0.01) | -0.11  (-0.20 to -0.03) | -0.18  (-0.26 to -0.09) | -0.21  (-0.29 to -0.13) | -0.04  (-0.15 to 0.08) | -0.1  (-0.22 to 0.02) | -0.13  (-0.25 to -0.02) |
| p value | 0.07 | 0.007 | <0.001 | <0.001 | 0.52 | 0.09 | 0.03 |
| **Secondary outcomes** |  |  |  |  |  |  |  |
| **Blood pressure, mm Hg** |  |  |  |  |  |  |  |
| Peripheral systolic |  |  |  |  |  |  |  |
| Baseline mean (SD) | 127.0 (11.3) | 128.3 (10.2) | 127.6 (10.7) | 128.3 (11.4) |  |  |  |
| Change at 6 months  (95% CI) | -2.4  (-4.3 to -0.5) | -2.9  (-4.7 to -1.0) | -0.9  (-2.8 to 1.1) | -2.7  (-4.5 to -0.8) | -0.5  (-3.2 to 2.2) | 1.6  (-1.1 to 4.2) | -0.2  (-2.9 to 2.5) |
| p value | 0.01 | 0.003 | 0.38 | 0.005 | 0.74 | 0.26 | 0.86 |
| Peripheral diastolic |  |  |  |  |  |  |  |
| Baseline mean (SD) | 79.5 (8.0) | 79.8 (7.2) | 80.0 (7.4) | 79.4 (7.2) |  |  |  |
| Change at 6 months  (95% CI) | -1.7  (-3.0 to -0.4) | -2.9  (-4.1 to -1.6) | -1.6  (-2.8 to -0.3) | -1.6  (-2.8 to -0.3) | -1.2  (-3.0 to 0.6) | 0.1  (-1.7 to 1.9) | 0.1  (-1.7 to 1.9) |
| p value | 0.01 | <0.001 | 0.02 | 0.01 | 0.19 | 0.88 | 0.91 |
| Central systolic |  |  |  |  |  |  |  |
| Baseline mean (SD) | 117.1 (10.8) | 118.5 (9.5) | 118.3 (10.2) | 118.4 (10.3) |  |  |  |
| Change at 6 months  (95% CI) | -1.9  (-3.5 to -0.2) | -2.2  (-3.8 to -0.6) | -0.7  (-2.5 to 1.0) | -1.9  (-3.5 to -0.3) | -0.3  (-2.7 to 2.0) | 1.1  (-1.3 to 3.6) | 0.0  (-2.4 to 2.3) |
| p value | 0.03 | 0.01 | 0.41 | 0.02 | 0.79 | 0.36 | 0.98 |
| Central diastolic |  |  |  |  |  |  |  |
| Baseline mean (SD) | 80.5 (7.9) | 80.6 (7.3) | 81.0 (7.4) | 80.3 (7.5) |  |  |  |
| Change at 6 months  (95% CI) | -1.9  (-3.3 to -0.6) | -2.9  (-4.1 to -1.6) | -1.5  (-2.8 to -0.2) | -1.6  (-2.9 to -0.3) | -0.9  (-2.7 to 0.9) | 0.4  (-1.4 to 2.3) | 0.3  (-1.5 to 2.2) |
| p value | 0.004 | <0.001 | 0.02 | 0.01 | 0.31 | 0.66 | 0.72 |
| **Cholesterol levels, mg/dL** |  |  |  |  |  |  |  |
| LDL cholesterol |  |  |  |  |  |  |  |
| Baseline mean (SD) | 123.2 (30.9) | 120.3 (28.7) | 122.5 (39.8) | 124.4 (27.8) |  |  |  |
| Change at 6 months  (95% CI) | -0.2  (-5.0 to 4.6) | 0.8  (-3.8 to 5.5) | -4.9  (-9.8 to -0.02) | -7.2  (-11.7 to -2.6) | 1.0  (-5.6 to 7.6) | -4.7  (-11.6 to 2.1) | -7.0  (-13.6 to -0.4) |
| p value | 0.94 | 0.72 | 0.049 | 0.002 | 0.76 | 0.18 | 0.04 |
| HDL cholesterol |  |  |  |  |  |  |  |
| Baseline mean (SD) | 48.8 (14.8) | 48.0 (15.2) | 46.7 (12.2) | 48.0 (14.2) |  |  |  |
| Change at 6 months  (95% CI) | 0.1  (-1.2 to 1.3) | 0.8  (-0.4 to 2.0) | 1.2  (0.0 to 2.4) | -0.2  (-1.4 to 1.0) | 0.7  (-1.0 to 2.5) | 1.1  (-0.7 to 2.9) | -0.3  (-2.0 to 1.5) |
| p value | 0.89 | 0.19 | 0.06 | 0.76 | 0.42 | 0.22 | 0.76 |
| **Fasting glucose, mg/dL** |  |  |  |  |  |  |  |
| Baseline mean (SD) | 95.8 (11.3) | 95.8 (9.9) | 95.9 (12.8) | 96.2 (12.5) |  |  |  |
| Change at 6 months  (95% CI) | -0.3  (-2.2 to 1.6) | 0.3  (-1.5 to 2.2) | -2.3  (-4.2 to -0.5) | 0.1  (-1.7 to 1.9) | 0.6  (-2.1 to 3.3) | -2.0  (-4.7 to 0.6) | 0.4  (-2.2 to 2.9) |
| p value | 0.77 | 0.72 | 0.02 | 0.93 | 0.65 | 0.13 | 0.79 |
| **Body composition** |  |  |  |  |  |  |  |
| Percent body fat, % |  |  |  |  |  |  |  |
| Baseline mean (SD) | 38.6 (7.0) | 38.5 (7.4) | 38.2 (7.2) | 37.9 (6.8) |  |  |  |
| Change at 6 months  (95% CI) | -0.3  (-0.7 to 0.1) | -1.1  (-1.5 to -0.7) | -1.1  (-1.5 to -0.7) | -1.5  (-1.9 to -1.1) | -0.8  (-1.3 to -0.3) | -0.8  (-1.4 to -0.3) | -1.2  (-1.8 to -0.7) |
| p value | 0.15 | <0.001 | <0.001 | <0.001 | 0.003 | 0.002 | <0.001 |
| Body weight, kg |  |  |  |  |  |  |  |
| Baseline mean (SD) | 91.4 (17.5) | 91.7 (18.1) | 90.4 (17.6) | 90.7 (17.8) |  |  |  |
| Change at 6 months  (95% CI) | 0.1  (-0.6 to 0.8) | -0.1  (-0.8 to 0.7) | -1.5  (-2.3 to -0.7) | -1.8  (-2.5 to -1.1) | -0.2  (-1.2 to 0.9) | -1.6  (-2.7 to -0.5) | -1.9  (-2.9 to -0.9) |
| p value | 0.80 | 0.86 | <0.001 | <0.001 | 0.76 | 0.003 | <0.001 |
| Waist circumference, cm |  |  |  |  |  |  |  |
| Baseline mean (SD) | 102.6 (12.3) | 103.3 (12.7) | 103.2 (12.4) | 102.3 (12.7) |  |  |  |
| Change at 6 months  (95% CI) | 0.8  (-0.4 to 2.0) | -0.4  (-1.6 to 0.8) | -1.8  (-3.0 to -0.5) | -1.7  (-2.9 to -0.4) | -1.2  (-2.9 to 0.5) | -2.5  (-4.3 to -0.7) | -2.5  (-4.2 to -0.7) |
| p value | 0.21 | 0.51 | 0.01 | 0.01 | 0.17 | 0.01 | 0.01 |
| Lean body mass, kg |  |  |  |  |  |  |  |
| Baseline mean (SD) | 52.4 (11.1) | 52.5 (11.1) | 52.2 (12.0) | 52.5 (11.2) |  |  |  |
| Change at 6 months  (95% CI) | 0.3  (-0.1 to 0.7) | 1.0  (0.6 to 1.4) | 0.0  (-0.4 to 0.3) | 0.2  (-0.1 to 0.6) | 0.7  (0.2 to 1.3) | -0.3  (-0.9 to 0.2) | 0.0  (-0.6 to 0.5) |
| p value | 0.17 | <0.001 | 0.81 | 0.22 | 0.01 | 0.25 | 0.86 |
| **Physical fitness** |  |  |  |  |  |  |  |
| VO_2peak_, mL/kg/min‡ |  |  |  |  |  |  |  |
| Baseline mean (SD) | 26.3 (6.6) | 25.5 (6.0) | 26.1 (6.2) | 26.5 (6.7) |  |  |  |
| Change at 6 months  (95% CI) | -0.6  (-1.3 to 0.2) | 0.3  (-0.4 to 1.1) | 1.7  (0.9 to 2.4) | 1.8  (1.1 to 2.6) | 0.9  (-0.2 to 2.0) | 2.2  (1.1 to 3.3) | 2.4  (1.3 to 3.5) |
| p value | 0.15 | 0.42 | <0.001 | <0.001 | 0.12 | <0.001 | <0.001 |
| 1RM chest press, kg§ |  |  |  |  |  |  |  |
| Baseline mean (SD) | 52.8 (22.2) | 57.1 (24.2) | 53.2 (23.8) | 54.6 (22.7) |  |  |  |
| Change at 6 months  (95% CI) | 0.1  (-1.5 to 1.6) | 8.3  (6.8 to 9.8) | -1.1  (-2.7 to 0.5) | 4.2  (2.7 to 5.7) | 8.2  (6.0 to 10.4) | -1.2  (-3.4 to 1.0) | 4.1  (1.9 to 6.3) |
| p value | 0.91 | <0.001 | 0.17 | <0.001 | <0.001 | 0.29 | <0.001 |
| 1RM leg press, kg§ |  |  |  |  |  |  |  |
| Baseline mean (SD) | 122.0 (49.9) | 126.5 (49.0) | 125.7 (53.5) | 125.7 (51.5) |  |  |  |
| Change at 6 months  (95% CI) | 6.7  (1.5 to 11.9) | 17.2  (12.1 to 22.4) | 1.8  (-3.4 to 7.1) | 12.8  (7.6 to 18.1) | 10.5  (3.2 to 17.9) | -4.9  (-12.3 to 2.6) | 6.1  (-1.3 to 13.5) |
| p value | 0.01 | <0.001 | 0.49 | <0.001 | 0.005 | 0.20 | 0.11 |

Baseline means (SDs) are the unadjusted values and the changes (95% CIs) from baseline to 6 months are the least-squares adjusted values including age, sex, and baseline value of each outcome as covariates in the intention-to-treat analyses including all 406 randomized participants.

*P values in between-group differences were calculated using linear mixed-effects models with repeated measures including age, sex, and baseline value of each outcome as covariates in the intention-to-treat analyses including all 406 randomized participants. †Composite CVD risk z-score is the mean of the sex-specific z-scores of four established CVD risk factors: resting systolic blood pressure, LDL cholesterol, fasting glucose, and percent body fat, with lower scores indicating lower overall CVD risk.

‡Peak oxygen consumption (VO_2peak_) was assessed during a maximal graded treadmill test using the Balke and Ware protocol. §One-repetition maximum (1RM) is the maximum weight a participant can lift in one attempt in the chest press or leg press.

| **Supplementary Table S2:** Effect of resistance, aerobic, and combined exercise on primary and secondary outcomes at 6 months and 1 year in per-protocol analyses^a^ | | | | | | | |
| --- | --- | --- | --- | --- | --- | --- | --- |
| **Outcome** | **Control**  **(n = 93)** | **Resistance**  **(n = 78)** | **Aerobic**  **(n = 63)** | **Combination**  **(n = 79)** | **Between-group difference (95% CI)*** | | |
|  |  |  |  |  | **Resistance**  **vs Control** | **Aerobic**  **vs Control** | **Combination**  **vs Control** |
| **Primary outcome** |  |  |  |  |  |  |  |
| **Composite CVD risk z-score**† |  |  |  |  |  |  |  |
| Baseline mean (SD) | -0.01 (0.56) | 0.04 (0.55) | -0.10 (0.56) | 0.00 (0.55) |  |  |  |
| Change at 6 months (95% CI) | -0.07  (-0.15 to 0.02) | -0.13  (-0.22 to -0.04) | -0.20  (-0.30 to -0.10) | -0.21  (-0.30 to -0.12) | -0.06  (-0.18 to 0.07) | -0.13  (-0.26 to 0.00) | -0.14  (-0.26 to -0.02) |
| p value | 0.11 | 0.01 | <0.001 | <0.001 | 0.35 | 0.05 | 0.03 |
| Change at 1 year (95% CI) | -0.01  (-0.09 to 0.08) | -0.07  (-0.16 to 0.02) | -0.22  (-0.33 to -0.12) | -0.19  (-0.28 to -0.10) | -0.07  (-0.19 to 0.06) | -0.22  (-0.35 to -0.09) | -0.18  (-0.31 to -0.06) |
| p value | 0.91 | 0.13 | <0.001 | <0.001 | 0.29 | 0.001 | 0.004 |
| **Secondary outcomes** |  |  |  |  |  |  |  |
| **Blood pressure, mm Hg** |  |  |  |  |  |  |  |
| Peripheral systolic |  |  |  |  |  |  |  |
| Baseline mean (SD) | 126.9 (11.2) | 129.1 (10.4) | 125.9 (10.0) | 128.6 (11.5) |  |  |  |
| Change at 6 months  (95% CI) | -2.5  (-4.3 to -0.6) | -3.2  (-5.2 to -1.3) | -1.1  (-3.3 to 1.1) | -2.9  (-4.9 to -0.9) | -0.8  (-3.5 to 1.9) | 1.3  (-1.6 to 4.2) | -0.5  (-3.2 to 2.2) |
| p value | 0.01 | 0.001 | 0.32 | 0.004 | 0.57 | 0.37 | 0.73 |
| Change at 1 year (95% CI) | -0.5  (-2.3 to 1.3) | -1.3  (-3.3 to 0.7) | -1.9  (-4.0 to 0.3) | -2.6  (-4.5 to -0.6) | -0.8  (-3.5 to 1.9) | -1.4  (-4.2 to 1.5) | -2.1  (-4.8 to 0.6) |
| p value | 0.60 | 0.20 | 0.10 | 0.01 | 0.56 | 0.35 | 0.13 |
| Peripheral diastolic |  |  |  |  |  |  |  |
| Baseline mean (SD) | 79.3 (8.0) | 80.1 (7.3) | 79.2 (7.2) | 79.2 (7.2) |  |  |  |
| Change at 6 months  (95% CI) | -1.6  (-2.8 to -0.3) | -3.1  (-4.5 to -1.8) | -1.6  (-3.1 to -0.1) | -1.8  (-3.1 to -0.4) | -1.6  (-3.4 to 0.3) | 0.0  (-1.9 to 2.0) | -0.2  (-2.1 to 1.6) |
| p value | 0.01 | <0.001 | 0.04 | 0.01 | 0.10 | 0.98 | 0.83 |
| Change at 1 year (95% CI) | -0.7  (-2.0 to 0.5) | -1.3  (-2.7 to 0.0) | -1.9  (-3.4 to -0.4) | -1.1  (-2.5 to 0.2) | -0.6  (-2.4 to 1.2) | -1.1  (-3.1 to 0.8) | -0.4  (-2.2 to 1.4) |
| p value | 0.24 | 0.05 | 0.01 | 0.10 | 0.53 | 0.25 | 0.68 |
| Central systolic |  |  |  |  |  |  |  |
| Baseline mean (SD) | 116.9 (10.6) | 119.1 (9.6) | 116.8 (9.8) | 118.6 (10.3) |  |  |  |
| Change at 6 months  (95% CI) | -1.8  (-3.5 to -0.2) | -2.6  (-4.3 to -0.8) | -1.0  (-3.0 to 1.0) | -2.0  (-3.8 to -0.2) | -0.7  (-3.1 to 1.7) | 0.9  (-1.7 to 3.4) | -0.1  (-2.6 to 2.3) |
| p value | 0.03 | 0.01 | 0.33 | 0.03 | 0.57 | 0.51 | 0.91 |
| Change at 1 year (95% CI) | -0.2  (-1.8 to 1.5) | -1.0  (-2.7 to 0.8) | -1.8  (-3.8 to 0.1) | -1.9  (-3.6 to -0.1) | -0.8  (-3.2 to 1.6) | -1.7  (-4.2 to 0.9) | -1.7  (-4.1 to 0.6) |
| p value | 0.85 | 0.28 | 0.07 | 0.03 | 0.50 | 0.20 | 0.15 |
| Central diastolic |  |  |  |  |  |  |  |
| Baseline mean (SD) | 80.3 (7.9) | 80.9 (7.4) | 80.1 (7.0) | 80.1 (7.5) |  |  |  |
| Change at 6 months  (95% CI) | -1.8  (-3.1 to -0.5) | -3.2  (-4.5 to -1.8) | -1.6  (-3.1 to -0.1) | -1.7  (-3.1 to -0.3) | -1.3  (-3.2 to 0.6) | 0.2  (-1.8 to 2.2) | 0.1  (-1.8 to 2.0) |
| p value | 0.01 | <0.001 | 0.04 | 0.01 | 0.17 | 0.82 | 0.94 |
| Change at 1 year (95% CI) | -0.9  (-2.2 to 0.4) | -1.2  (-2.6 to 0.2) | -2.0  (-3.5 to -0.5) | -1.1  (-2.4 to 0.3) | -0.3  (-2.1 to 1.6) | -1.1  (-3.0 to 0.9) | -0.2  (-2.0 to 1.7) |
| p value | 0.16 | 0.09 | 0.01 | 0.11 | 0.76 | 0.29 | 0.85 |
| **Cholesterol levels, mg/dL** |  |  |  |  |  |  |  |
| LDL cholesterol |  |  |  |  |  |  |  |
| Baseline mean (SD) | 124.2 (30.8) | 118.4 (28.1) | 118.7 (44.6) | 122.7 (26.4) |  |  |  |
| Change at 6 months  (95% CI) | -0.6  (-5.6 to 4.4) | 0.6  (-4.8 to 6.0) | -5.0  (-11.0 to 0.9) | -7.3  (-12.6 to -2.0) | 1.2  (-6.1 to 8.6) | -4.4  (-12.2 to 3.4) | -6.7  (-14.0 to 0.6) |
| p value | 0.82 | 0.82 | 0.10 | 0.01 | 0.75 | 0.26 | 0.07 |
| Change at 1 year (95% CI) | 0.5  (-6.2 to 7.1) | -0.5  (-7.7 to 6.7) | -5.4  (-13.5 to 2.6) | -6.6  (-13.8 to 0.6) | -0.9  (-10.7 to 8.9) | -5.9  (-16.3 to 4.5) | -7.1  (-16.9 to 2.7) |
| p value | 0.89 | 0.90 | 0.18 | 0.07 | 0.85 | 0.27 | 0.16 |
| HDL cholesterol |  |  |  |  |  |  |  |
| Baseline mean (SD) | 48.8 (15.4) | 48.7 (15.5) | 46.7 (12.4) | 47.8 (13.9) |  |  |  |
| Change at 6 months  (95% CI) | 0.1  (-1.1 to 1.3) | 0.6  (-0.7 to 2.0) | 1.3  (-0.2 to 2.8) | 0.4  (-0.9 to 1.7) | 0.5  (-1.3 to 2.4) | 1.2  (-0.7 to 3.1) | 0.3  (-1.5 to 2.1) |
| p value | 0.87 | 0.35 | 0.08 | 0.54 | 0.57 | 0.22 | 0.73 |
| Change at 1 year (95% CI) | -0.7  (-2.0 to 0.6) | 1.2  (-0.3 to 2.6) | 1.5  (-0.1 to 3.1) | 2.0  (0.5 to 3.4) | 1.9  (-0.1 to 3.8) | 2.2  (0.1 to 4.3) | 2.7  (0.7 to 4.6) |
| p value | 0.29 | 0.11 | 0.07 | 0.01 | 0.06 | 0.04 | 0.007 |
| **Fasting glucose, mg/dL** |  |  |  |  |  |  |  |
| Baseline mean (SD) | 95.5 (10.4) | 95.6 (9.5) | 96.2 (14.3) | 96.5 (12.4) |  |  |  |
| Change at 6 months  (95% CI) | 0.0  (-1.9 to 2.0) | 0.5  (-1.6 to 2.5) | -2.4  (-4.7 to -0.1) | 0.6  (-1.5 to 2.6) | 0.4  (-2.4 to 3.3) | -2.5  (-5.5 to 0.6) | 0.5  (-2.3 to 3.4) |
| p value | 0.98 | 0.65 | 0.04 | 0.60 | 0.76 | 0.11 | 0.71 |
| Change at 1 year (95% CI) | 0.3  (-1.3 to 1.9) | 1.7  (-0.1 to 3.4) | -2.5  (-4.4 to -0.5) | 0.4  (-1.3 to 2.2) | 1.4  (-1.0 to 3.8) | -2.8  (-5.3 to -0.3) | 0.2  (-2.2 to 2.5) |
| p value | 0.73 | 0.06 | 0.01 | 0.63 | 0.26 | 0.03 | 0.90 |
| **Body composition** |  |  |  |  |  |  |  |
| Percent body fat, % |  |  |  |  |  |  |  |
| Baseline mean (SD) | 38.2 (7.0) | 38.2 (7.5) | 37.7 (7.0) | 37.7 (6.8) |  |  |  |
| Change at 6 months  (95% CI) | -0.2  (-0.6 to 0.1) | -1.2  (-1.6 to -0.8) | -1.4  (-1.8 to -0.9) | -1.6  (-2.0 to -1.2) | -1.0  (-1.5 to -0.4) | -1.1  (-1.7 to -0.6) | -1.4  (-1.9 to -0.8) |
| p value | 0.23 | <0.001 | <0.001 | <0.001 | <0.001 | <0.001 | <0.001 |
| Change at 1 year (95% CI) | -0.1  (-0.5 to 0.2) | -1.3  (-1.6 to -0.9) | -1.6  (-2.0 to -1.2) | -1.4  (-1.8 to -1.1) | -1.2  (-1.7 to -0.6) | -1.5  (-2.0 to -0.9) | -1.3  (-1.8 to -0.8) |
| p value | 0.54 | <0.001 | <0.001 | <0.001 | <0.001 | <0.001 | <0.001 |
| Body weight, kg |  |  |  |  |  |  |  |
| Baseline mean (SD) | 91.6 (17.8) | 92.4 (19.1) | 88.6 (16.6) | 90.7 (17.3) |  |  |  |
| Change at 6 months  (95% CI) | 0.1  (-0.7 to 0.8) | -0.2  (-1.0 to 0.6) | -2.1  (-3.0 to -1.2) | -2.1  (-2.8 to -1.3) | -0.3  (-1.4 to 0.8) | -2.2  (-3.3 to -1.0) | -2.1  (-3.2 to -1.1) |
| p value | 0.85 | 0.60 | <0.001 | <0.001 | 0.60 | <0.001 | <0.001 |
| Change at 1 year (95% CI) | -0.2  (-1.1 to 0.6) | 0.3  (-0.7 to 1.2) | -2.2  (-3.2 to -1.2) | -1.8  (-2.7 to -0.9) | 0.5  (-0.8 to 1.7) | -2.0  (-3.3 to -0.6) | -1.6  (-2.8 to -0.4) |
| p value | 0.61 | 0.57 | <0.001 | <0.001 | 0.45 | 0.003 | 0.01 |
| Waist circumference, cm |  |  |  |  |  |  |  |
| Baseline mean (SD) | 102.3 (12.5) | 103.2 (13.0) | 102.0 (12.5) | 102.3 (11.9) |  |  |  |
| Change at 6 months  (95% CI) | 0.8  (-0.4 to 2.0) | -0.7  (-2.0 to 0.6) | -2.3  (-3.8 to -0.9) | -1.9  (-3.2 to -0.6) | -1.5  (-3.3 to 0.2) | -3.1  (-5.0 to -1.2) | -2.7  (-4.5 to -0.9) |
| p value | 0.19 | 0.28 | 0.002 | 0.004 | 0.09 | 0.001 | 0.003 |
| Change at 1 year (95% CI) | -0.7  (-1.9 to 0.6) | -1.2  (-2.5 to 0.1) | -3.5  (-5.0 to -2.0) | -3.3  (-4.6 to -2.0) | -0.5  (-2.4 to 1.3) | -2.8  (-4.8 to -0.9) | -2.6  (-4.5 to -0.8) |
| p value | 0.29 | 0.08 | <0.001 | <0.001 | 0.56 | 0.004 | 0.004 |
| Lean body mass, kg |  |  |  |  |  |  |  |
| Baseline mean (SD) | 52.8 (11.3) | 53.0 (11.3) | 51.6 (11.3) | 52.7 (11.2) |  |  |  |
| Change at 6 months  (95% CI) | 0.2  (-0.2 to 0.6) | 1.0  (0.6 to 1.4) | -0.1  (-0.6 to 0.3) | 0.1  (-0.3 to 0.5) | 0.8  (0.2 to 1.3) | -0.3  (-0.9 to 0.3) | -0.1  (-0.6 to 0.5) |
| p value | 0.26 | <0.001 | 0.62 | 0.55 | 0.007 | 0.27 | 0.73 |
| Change at 1 year (95% CI) | -0.1  (-0.5 to 0.4) | 1.3  (0.8 to 1.7) | 0.0  (-0.5 to 0.5) | 0.2  (-0.3 to 0.6) | 1.3  (0.7 to 1.9) | 0.1  (-0.6 to 0.7) | 0.3  (-0.4 to 0.9) |
| p value | 0.74 | <0.001 | >0.99 | 0.39 | <0.001 | 0.83 | 0.40 |
| **Physical fitness** |  |  |  |  |  |  |  |
| VO_2peak_, mL/kg/min‡ |  |  |  |  |  |  |  |
| Baseline mean (SD) | 26.7 (6.5) | 25.3 (5.9) | 26.7 (6.1) | 27.0 (6.8) |  |  |  |
| Change at 6 months  (95% CI) | -0.8  (-1.5 to 0.0) | 0.6  (-0.3 to 1.4) | 2.4  (1.5 to 3.3) | 2.1  (1.3 to 2.9) | 1.3  (0.2 to 2.4) | 3.1  (2.0 to 4.3) | 2.9  (1.8 to 4.0) |
| p value | 0.05 | 0.18 | <0.001 | <0.001 | 0.02 | <0.001 | <0.001 |
| Change at 1 year (95% CI) | -1.2  (-2.1 to -0.3) | 0.9  (-0.1 to 1.9) | 3.5  (2.4 to 4.6) | 2.3  (1.3 to 3.3) | 2.1  (0.8 to 3.4) | 4.7  (3.3 to 6.1) | 3.5  (2.2 to 4.9) |
| p value | 0.01 | 0.07 | <0.001 | <0.001 | 0.002 | <0.001 | <0.001 |
| 1RM chest press, kg§ |  |  |  |  |  |  |  |
| Baseline mean (SD) | 53.6 (22.4) | 57.2 (22.8) | 52.4 (20.7) | 55.2 (22.7) |  |  |  |
| Change at 6 months  (95% CI) | 0.1  (-1.4 to 1.5) | 9.2  (7.6 to 10.8) | 0.0  (-1.7 to 1.8) | 4.9  (3.3 to 6.4) | 9.2  (7.0 to 11.3) | -0.1  (-2.4 to 2.2) | 4.8  (2.6 to 7.0) |
| p value | 0.92 | <0.001 | >0.99 | <0.001 | <0.001 | 0.95 | <0.001 |
| Change at 1 year (95% CI) | -0.4  (-2.0 to 1.2) | 12.2  (10.5 to 14.0) | -0.8  (-2.7 to 1.2) | 8.0  (6.2 to 9.7) | 12.6  (10.3 to 15.0) | -0.4  (-2.9 to 2.2) | 8.4  (6.0 to 10.7) |
| p value | 0.62 | <0.001 | 0.44 | <0.001 | <0.001 | 0.78 | <0.001 |
| 1RM leg press, kg§ |  |  |  |  |  |  |  |
| Baseline mean (SD) | 123.7 (50.6) | 124.7 (45.9) | 121.3 (49.8) | 126.3 (54.2) |  |  |  |
| Change at 6 months  (95% CI) | 6.6  (1.4 to 11.7) | 19.1  (13.5 to 24.7) | 2.9  (-3.3 to 9.1) | 16.1  (10.5 to 21.6) | 12.5  (4.9 to 20.1) | -3.7  (-11.7 to 4.4) | 9.5  (1.9 to 17.1) |
| p value | 0.01 | <0.001 | 0.36 | <0.001 | 0.002 | 0.37 | 0.01 |
| Change at 1 year (95% CI) | 4.9  (-0.6 to 10.4) | 27.5  (21.5 to 33.5) | 5.5  (-1.2 to 12.1) | 16.2  (10.3 to 22.1) | 22.6  (14.4 to 30.7) | 0.5  (-8.0 to 9.1) | 11.3  (3.2 to 19.3) |
| p value | 0.08 | <0.001 | 0.11 | <0.001 | <0.001 | 0.90 | 0.006 |

Baseline means (SDs) are the unadjusted values and the changes (95% CIs) from baseline to 6 months or 1 year are the least-squares adjusted values including age, sex, and baseline value of each outcome as covariates. Per-protocol analysis includes participants who completed the study (excluding dropouts) and attended at least 80% of their prescribed exercise session over 1 year in exercise participants.

*P values in between-group differences were calculated using linear mixed-effects models with repeated measures including age, sex, and baseline value of each outcome as covariates. In a Bonferroni correction to adjust for the multiple comparisons in the composite CVD risk z-score at 1 year (primary outcome endpoint), the corrected p values were 0.87 for the resistance vs control group, 0.003 for the aerobic vs control group, and 0.012 for the combination vs control group.

†Composite CVD risk z-score is the mean of the sex-specific z-scores of four established CVD risk factors: resting systolic blood pressure, LDL cholesterol, fasting glucose, and percent body fat, with lower scores indicating lower overall CVD risk.

‡Peak oxygen consumption (VO_2peak_) was assessed during a maximal graded treadmill test using the Balke and Ware protocol. §One-repetition maximum (1RM) is the maximum weight a participant can lift in one attempt in the chest press or leg press.

| **Supplementary Table S3:** Adverse events* | | | | |
| --- | --- | --- | --- | --- |
| **Adverse event** | **Control**  **(n = 102)** | **Resistance**  **(n = 102)** | **Aerobic**  **(n = 101)** | **Combination**  **(n = 101)** |
| Tripped and fell right after treadmill exercise testing | 0 | 1 | 0 | 0 |
| Right shoulder pain | 0 | 0 | 0 | 1 |
| Right hip pain | 0 | 0 | 1 | 0 |
| Fainted right after exercise due to dehydration | 0 | 0 | 0 | 1 |
| Total | 0 | 1 | 1 | 2 |

*Number of participants with each reported adverse event across the study groups. There were no significant differences in total adverse events between the groups (p=0.56). Included are all reported adverse events that occurred during the intervention period. Participants may have had other adverse events that were not reported to the study staff.

**Supplementary Figure S1:** Changes in the CVD risk factors from baseline to 6 months

Results are from the linear mixed-effects models with repeated measures in the intention-to-treat analyses including all 406 randomized participants. Figure shows mean changes in the z-scores of the composite CVD risk factors by sex and age groups (A) and mean changes in the standardized z-scores of the individual and composite CVD risk factors (B) from baseline to 6 months. Z-score values below 0 indicate favorable changes in CVD risk factors. Whiskers indicate 95% confidence intervals (CIs). CVD=cardiovascular disease. LDL=low-density lipoprotein.
